# The Genome-Wide Interaction Network of Nutrient Stress Genes in *Escherichia coli*

**DOI:** 10.1128/mBio.01714-16

**Published:** 2016-11-22

**Authors:** Jean-Philippe Côté, Shawn French, Sebastian S. Gehrke, Craig R. MacNair, Chand S. Mangat, Amrita Bharat, Eric D. Brown

**Affiliations:** Michael G. DeGroote Institute for Infectious Disease Research, Department of Biochemistry and Biomedical Sciences, McMaster University, Hamilton, Ontario, Canada

## Abstract

Conventional efforts to describe essential genes in bacteria have typically emphasized nutrient-rich growth conditions. Of note, however, are the set of genes that become essential when bacteria are grown under nutrient stress. For example, more than 100 genes become indispensable when the model bacterium *Escherichia coli* is grown on nutrient-limited media, and many of these nutrient stress genes have also been shown to be important for the growth of various bacterial pathogens *in vivo*. To better understand the genetic network that underpins nutrient stress in *E. coli*, we performed a genome-scale cross of strains harboring deletions in some 82 nutrient stress genes with the entire *E. coli* gene deletion collection (Keio) to create 315,400 double deletion mutants. An analysis of the growth of the resulting strains on rich microbiological media revealed an average of 23 synthetic sick or lethal genetic interactions for each nutrient stress gene, suggesting that the network defining nutrient stress is surprisingly complex. A vast majority of these interactions involved genes of unknown function or genes of unrelated pathways. The most profound synthetic lethal interactions were between nutrient acquisition and biosynthesis. Further, the interaction map reveals remarkable metabolic robustness in *E. coli* through pathway redundancies. In all, the genetic interaction network provides a powerful tool to mine and identify missing links in nutrient synthesis and to further characterize genes of unknown function in *E. coli*. Moreover, understanding of bacterial growth under nutrient stress could aid in the development of novel antibiotic discovery platforms.

## IMPORTANCE

With the rise of antibiotic drug resistance, there is an urgent need for new antibacterial drugs. Here, we studied a group of genes that are essential for the growth of *Escherichia coli* under nutrient limitation, culture conditions that arguably better represent nutrient availability during an infection than rich microbiological media. Indeed, many such nutrient stress genes are essential for infection in a variety of pathogens. Thus, the respective proteins represent a pool of potential new targets for antibacterial drugs that have been largely unexplored. We have created all possible double deletion mutants through a genetic cross of nutrient stress genes and the *E. coli* deletion collection. An analysis of the growth of the resulting clones on rich media revealed a robust, dense, and complex network for nutrient acquisition and biosynthesis. Importantly, our data reveal new genetic connections to guide innovative approaches for the development of new antibacterial compounds targeting bacteria under nutrient stress.

## INTRODUCTION

The genome of *Escherichia coli* K-12 contains about 4,300 genes, but only 303 of these are considered to be essential (1, 2). Essential genes are conventionally defined as those required for growth under optimal conditions, and in *E. coli*, they are well documented (1, 3). Essentiality is, however, highly dependent on genetic and environmental context.

Even within the set of conventional essential genes, there are some that do not encode typical housekeeping functions and can be deleted in the right genetic context (4). Toxin-antitoxin system genes, for example, encode both lethal toxins and antitoxins to prevent self-intoxication (5). Here, the antitoxin gene has an essential phenotype but becomes dispensable in a strain where the toxin gene has been deleted. This type of genetic interaction is a synthetic viable interaction (6). Alternatively, synthetic lethal interactions occur when the combined deletion of two otherwise dispensable genes leads to a nonviable phenotype (7). For instance, parallel chaperone pathways in the periplasm, encoded by *surA* and *skp* and *degP*, carry outer membrane proteins to the outer membrane of *E. coli* (8). Deletion of either gene produces perfectly viable cells, while deletion of both is lethal. Other examples of synthetic lethality are found in various aspects of bacterial physiology such as DNA damage and repair (9), cell division (10), outer membrane biogenesis (11), and metabolism (12). It is worth noting that synthetic interactions often involve genes that are not linked on the chromosome and that are not related to each other. Overall, these examples highlight instances where gene essentiality is highly dependent on genetic context.

The growth environment also affects gene dispensability. Indeed, scores of genes resident in common bacterial pathogens are essential for infection *in vivo* but are dispensable when cultured *in vitro* (13–17). Furthermore, when *E. coli* is grown in nutrient-limited media, more than 100 genes become essential (1, 18, 19), principally those required for the synthesis of amino acids, vitamins, and nucleobases. Interestingly, the sets of *in vivo* essential and nutrient stress genes show considerable overlap (13, 16, 17). Of note, Jorth et al. (16) recently probed genes involved in metabolism during the infection process and found that many nutrient stress genes, involved in biotin, pantothenate, glycine, and tyrosine metabolism among others, contribute to pathogen fitness *in vivo*. Moreover, for the pathogen *Mycobacterium tuberculosis*, the synthesis of certain vitamins is crucial for the establishment of an infection (13, 20), and this has prompted several groups to look for inhibitors of biotin and pantothenate biosynthesis (21).

In all, the environmental context of nutrient stress may well be a better proxy for the conditions during an infection than rich microbiological media. Naturally, this expands the list of potential targets for antimicrobial therapies and facilitates whole-cell screening and target discovery platforms that make use of suppression by nutrients (22, 23). These efforts are helped by many decades of study of bacterial physiology that have yielded an extensive understanding of individual enzymes and their biosynthetic pathways. Nevertheless, relatively little is known about the interactions of nutrient stress genes and their connections more broadly to functions encoded in that fraction of the genome that is not conventionally associated with nutrient stress. For example, the mechanistic basis for the synergistic interaction between trimethoprim and sulfamethoxazole, a synergistic antibiotic combination that impinges on folate biosynthesis and has been widely used for decades, remains poorly understood (24). Indeed, genetic networks that underpin nutrient biosynthesis in bacteria have largely not been probed thus far. Here, we describe an effort to cross the set of genes necessary for the growth of the model microbe *E. coli* on nutrient-limited media with all mutants in the comprehensive gene deletion collection (Keio) (1). We have analyzed growth of the resulting double deletion mutants on rich microbiological media, allowing us to identify hitherto unknown connections in biosynthesis pathways and to link functions to previously uncharacterized genes. Our data highlight a surprising number and density of genetic interactions inherent in nutrient biosynthesis, including important redundancy to buffer perturbations associated with nutrient stress.

## RESULTS

### Synthetic genetic array of nutrient stress genes.

**FIG 1:**
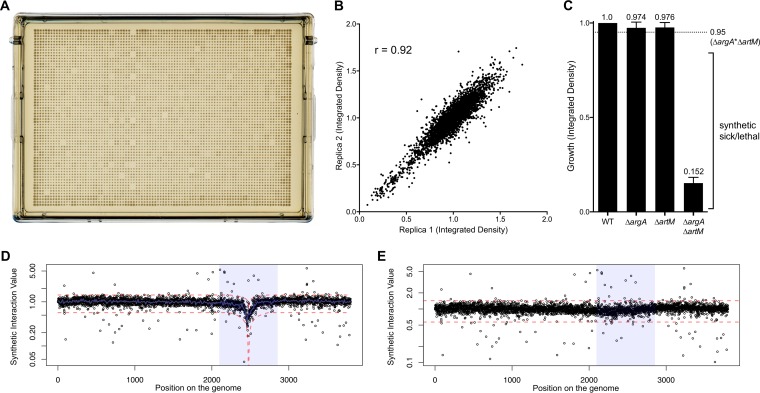
High-throughput array to detect synthetic sick and lethal interactions. Shown here is an example of data from the mating of the *argA* deletion mutant with strains of the *E. coli* (Keio) deletion collection. (A) Example of a selection plate that contains 1,536 double deletion mutants in quadruplicate to give a total of 6,144 colonies per plate. (B) Replica plot of the integrated densities of two biological duplicates of the cross of the *argA* deletion mutant with the Keio collection. (C) Multiplicative approach to detect synthetic sick or lethal interactions. The growth of the single deletion and double deletion mutants are relative to that of wild-type (WT) *E. coli* strain BW25113. The dotted line delineates the expected growth defect from the accumulation of the single deletions as described in detail in Materials and Methods. (D) Index plot showing the synthetic interaction value of every double deletion mutant. (E) Correction of the dip using a rolling median as described in Materials and Methods.

In *E. coli*, 119 genes become essential when cells are grown in nutrient-limited media. In order to better understand gene essentiality during nutrient stress, we crossed bacteria with single gene deletions of these 119 genes with mutants in the genome-scale single deletion set (Keio) using synthetic genetic array methodology (25, 26). The approach relies on the high-throughput engineering of double deletion mutants by bacterial conjugation, where a query gene deletion is combined with every single gene deletion mutant in the Keio collection (see Fig. S1 in the supplemental material). We conducted the conjugation on plates containing 1,536 colonies and transferred each colony in quadruplicate onto the selection plates to obtain 6,144 colonies per plate (Fig. 1A and Fig. S1). Finally, we monitored the growth of every double deletion mutant over 18 h using the method of French et al. (27).

Our synthetic genetic array analysis was performed in biological duplicates. The data were of high quality as evidenced by the correlation of replicates (Fig. 1B). Synthetic genetic arrays give information about synthetic sick/lethal gene pairs that are defined by a growth defect that is worse than what is expected from the accumulation of the single deletions alone. Such interactions are defined by the so-called multiplicative rule (7, 28), where the expected growth is the product of the growth defects seen for the individual genes. As an example, *argA* and *artM* formed a synthetic lethal pair, as the relative growth of the double deletion mutants was significantly less than the expected growth (Fig. 1C). As also noted previously by others using synthetic genetic arrays (25, 26), we have observed a significant effect of the distance between the position of the query gene deletion and the Keio deletion on the growth of the double mutant. Indeed, ordering the Keio clones according to their position on the chromosome created a dip in the index plot around the position of the query deletion (Fig. 1D). This dip is thought to be an artifact of the recombination process. In the case of closely linked genes, it is possible that the efficiency of recombination was not optimal or that the recombination event excluded the kanamycin cassette from the recipient strain (29). To correct for this dip, we modeled the region flanking the query gene. Symmetrical logarithmic curves were fit to the rolling median of the data in the region of the query gene. The data are subsequently standardized to 1, by offsetting by the value of the fit (Fig. 1E). We confirmed the accuracy of this novel correction method by reconstructing several double deletion mutants from corrected regions (see Fig. S3 in the supplemental material).

Other genes also affected the conjugation and recombination processes. For instance, *recA* formed synthetic lethal interactions with every query gene, likely because the recipient cells are deficient for recombination (30). Furthermore, some strains with mutations in envelope biogenesis genes, such as *rfaJ*, *fabH*, *wecB*, or *cpxA*, frequently formed synthetic sick or lethal interactions. This may have been due to an inefficient mating procedure (31, 32). Interestingly, we have also observed that the conjugation process was less efficient in deletion mutants displaying a morphological defect (French et al., unpublished data).

**FIG 2:**
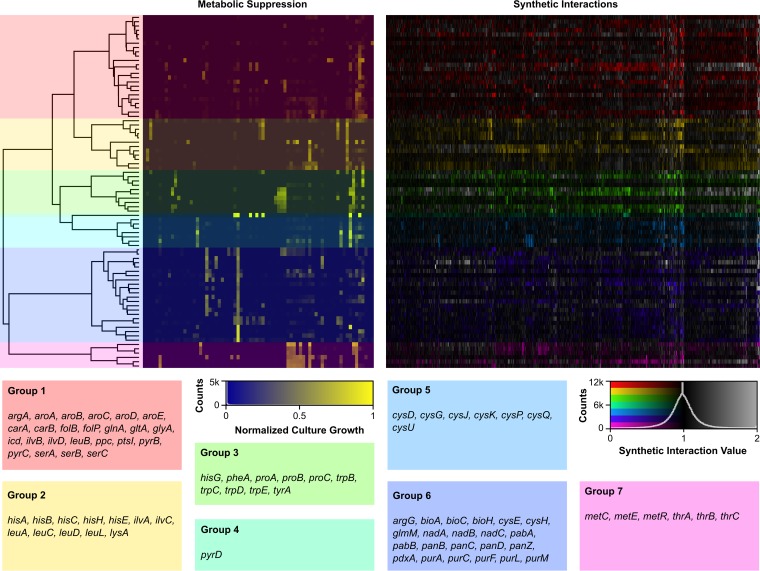
Synthetic genetic array of nutrient stress genes. Metabolite suppression profiles (left) were clustered using Ward’s least variance and used to order the synthetic interaction profiles for 82 of the nutrient-limited essential genes (right). The dendrogram was divided to yield seven distinct groupings. These groups can be generally simplified in biosynthesis function as amino acids, folate and pyrimidine biosynthesis (group 1), nonaromatic hydrophobic-side-chain amino acid (group 2), aromatic amino acids (group 3), pyrimidines (group 4), cysteine (group 5), purines and vitamins (group 6), and threonine and methionine (group 7). This was done to group on the basis of metabolic responses to nutrient limitation and look for synthetic lethal interactions common to similar suppression profiles.

We obtained quality data for 82 of the 119 nutrient stress genes, spanning most pathways (Fig. 2; see Table S2 in the supplemental material). In the remaining genes, the conjugation between the query deletion strain and the Keio clones was not efficient, resulting in unreliable data that were discarded from our analysis. In total, across 315,400 double deletion mutants, we identified 1,881 synthetic sick or lethal interactions (Fig. 2; Table S2). This corresponds to an average of 23 interactions per nutrient stress gene.

Some double deletion mutants also grew better than expected by the multiplicative rule of synthetic interactions (Fig. 2). These mutants represent beneficial interactions where a second deletion suppresses the growth defect of the first mutation. Many of these beneficial interactions were generalized across the nutrient stress genes, such as the *ptsH* gene or the *aceE* gene, while others were more specific to different pathways. For instance, all the tryptophan biosynthesis genes formed a beneficial interaction with the uncharacterized gene *yhdU*. Overall, beneficial interactions were not as prevalent and as informative as synthetic sick and lethal interactions. Therefore, we have focused our analysis herein on synthetic sick and lethal interactions.

Each nutrient stress gene was also subjected to the metabolic suppression array of Zlitni et al. (22) in order to define metabolic functional similarities between the genes. The metabolite suppression array is a 96-test condition supplementation system where cells are grown in M9 minimal medium and in the presence of added nutrients or pools thereof to define the nature of the auxotrophy that is generated by the deletion or with inhibitors of nutrient biosynthesis (see Text S1 and Table S3 in the supplemental material). The nutrient stress genes were then clustered based on their metabolic suppression profiles. A dendrogram was generated from metabolic suppression profiles, defining seven groups that were used to cluster the genetic interaction array (Fig. 2). This clustering method separated amino acid biosynthesis from vitamin and purine biosynthesis (group 6) and aromatic amino acid biosynthesis (group 3). This method further clustered genes involved in the biosynthesis of similar amino acids. Indeed, branched-chain amino acid biosynthesis genes (group 2) and cysteine biosynthesis genes (group 5) were grouped together, as well as methionine and threonine, two amino acids linked to the biosynthesis of homoserine (group 7). Clustering in this manner, based on biological response rather than synthetic interaction, allowed us to identify trends in synthetic lethal interactions between groups with similar metabolic profiles. Of note, genes encoding the putative Sap ABC transporter *sapB*, *sapC*, and *sapD* were synthetic lethal with group 3 members, suggesting that the Sap transporter might be involved in aromatic amino acid metabolism. Members of group 2 formed unique synthetic lethal interactions with *astDE*, two genes involved in arginine catabolism (33).

**FIG 3:**
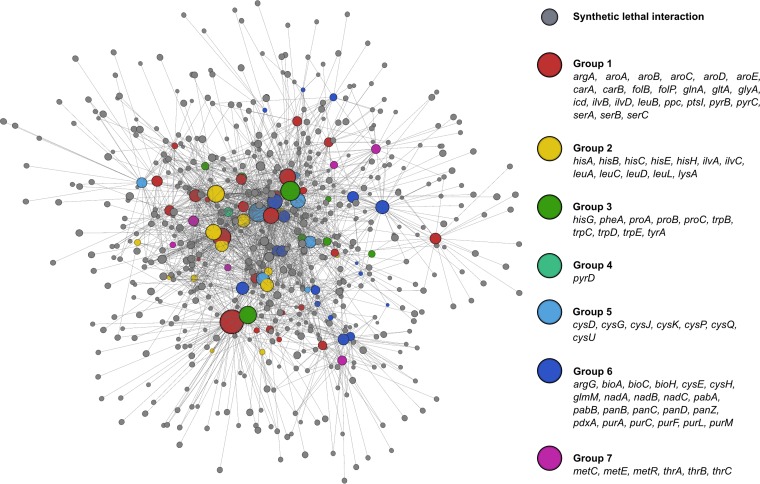
Genetic interaction network for nutrient stress genes**.** Synthetic lethal interactions are shown here, with nutrient stress gene nodes colored according to their groupings from Fig. 2. The three-dimensional (3D) network was generated using BioLayout Express3D (1), with nutrient stress nodes sized according to their number of edges.

Synthetic sick and lethal interactions were used to generate a complex network map (Fig. 3; see Table S2 in the supplemental material), showing high neighborhood connectivity among nutrient stress genes. This was especially true within most of the individual groupings. In fact, there were only 254 genes that interacted uniquely with nutrient stress genes, while the remaining 1,627 had more than one connecting edge.

### Profound interactions between transport and biosynthesis.

Our gene-gene interaction data highlighted different types of interactions. The first category, and by far the largest one, included interactions with a gene of unknown mechanism. Many interactions were recorded with uncharacterized genes or between unrelated genes. While this type of interaction is of much interest, it is hard to predict the reason behind the observed synthetic lethality. This emphasizes that there is much more to understand behind the physiology of bacteria. Indeed, even in a set of genes that is relatively well characterized such as these nutrient stress genes, most of the observed interactions occurred with partners that would not have been suspected otherwise. Of note, Gene Ontology (GO) term analysis of our set of synthetic sick and lethal interactions was enriched in terms related to transport, cellular metabolic processes, and oxidation-reduction processes, including many NADH-dependent processes (see Fig. S4 in the supplemental material). This suggests that nutrient biosynthesis is extensively linked to the central metabolism of the cell. Overall, our data highlight a previously unseen complexity in nutrient metabolism.

Our data also highlighted the redundancy in the genome of *E. coli* dedicated to the acquisition and synthesis of amino acids and nucleotides. For instance, the biosynthesis of pantothenate is essential during growth in nutrient-limited conditions (see Fig. S5 in the supplemental material). In our synthetic genetic array, the genes involved in the biosynthesis of pantothenate (*panB*, *panC*, *panD*, and *panZ*) are involved in about 100 synthetic sick or lethal interactions. However, only four genes formed synthetic lethal interactions with all genes involved in pantothenate biosynthesis: *recA*, *recC*, *ydhT*, and *panF* (Fig. S5A). As indicated above, *recA* and *recC* showed a synthetic lethal phenotype because conjugation/recombination was not efficient in these mutants. The other two genes, *panF* and *yhdT*, overlap by 10 nucleotides, suggesting that the deletion in *yhdT* likely also disrupts *panF*. Interestingly, *panF* is the transporter for pantothenate (Fig. S5B). Therefore, the only gene that formed synthetic lethal interactions with all pantothenate biosynthesis genes is the transporter *panF*. To confirm that *panF* was specifically interacting with the pantothenate biosynthesis genes, we created an apramycin-resistant *panF* deletion mutant that we subsequently crossed with the Keio collection. As expected, a *panF* deletion mutant formed synthetic lethal interactions only with genes that affected conjugation/recombination and with the genes involved in pantothenate biosynthesis (Fig. S5C). Taken together, these results validate that our genetic interaction network can identify authentic synthetic lethal interactions. Furthermore, these results demonstrate that pantothenate biosynthesis genes are dispensable when bacteria can acquire pantothenate from the media but that these genes are essential when no extracellular pantothenate is available. Pantothenate biosynthesis represents the first step toward the biosynthesis of coenzyme A, and genes involved in the further transformation of pantothenate into coenzyme A are essential (34). It is, therefore, not surprising that when bacteria lose their ability to import pantothenate from the extracellular media, the biosynthesis of pantothenate becomes essential even in nutrient-rich conditions.

We have observed this type of interaction, between biosynthetic and transport genes, in other instances (Table 1). In many cases where there are no interactions between transport and biosynthesis, there is usually more than one transporter that can import the metabolites. For example, genes involved in leucine and isoleucine did not interact with any transporter. Both of these amino acids can be imported by the same branched-chain amino acid transporter BrnQ (35), but alternate systems can also transport these branched-chain amino acids (36). As such, the deletion of a single transport gene is sometimes insufficient to render nutrient biosynthetic enzymes essential in rich media.

**Table 1:** Synthetic interactions highlighting pathway redundancy

Gene	Partner	Function	Type(s) of interaction(s)
*argA*	*artM*, *artP*, or *artQ*	Subunit of arginine transporter	Biosynthesis and transport
*argG*			
*aroA*	*tyrP*	Tyrosine transporter	Biosynthesis and transport
*aroC*			
*tyrA*			
*panB*	*panF*	Pantothenate transporter	Biosynthesis and transport
*panC*			
*panD*			
*panZ*			
*glnA*	*glnQ*, *glnP*, or *glnH*	Glutamine ABC transporter	Biosynthesis and transport
*icd*			
*lysA*	*lysP*	Lysine transporter	Biosynthesis and transport
*gltA*	*gltI*, *gltJ*, *gltL*, or *gltK*	Glutamate ABC transporter	Biosynthesis and transport
*thrA*	*metL*	Aspartate kinase/homoserine dehydrogenase	Redundant enzyme
*nadA*	*pncB*	Nicotinate phosphoribosyltransferase	Redundant pathways
*nadB*			
*nadC*			
*glyA*	*gcvHPT*	Glycine cleavage system	Redundant pathways
*glyA*	*gcvA*	Glycine cleavage system activator	Redundant pathways
*glyA*	*lrp*	Leucine-responsive transcriptional regulator	Redundant pathways

We also observed synthetic lethal interactions between pairs of genes that are redundant and lead to the biosynthesis of the same metabolite (Table 1). For instance, *metL* and *thrA* are involved in the biosynthesis of methionine and threonine, respectively, consistent with suppression of their growth phenotypes by these amino acids evident in the metabolic suppression array (see Table S3 in the supplemental material). Both enzymes possess the same enzymatic activity and are involved in the biosynthesis of homoserine, a precursor of methionine and threonine. The homoserine biosynthesis pathway is indispensable for the growth of *E. coli* on rich media, as exemplified by the fact that the *asd* gene has an essential phenotype (37). Indeed, our data suggest that this is the case, as *metL* and *thrA* formed a synthetic lethal gene pair. Another notable interaction in this category of redundant pathways was the *glyA* gene with the genes that code for the subunits of the glycine cleavage system (*gcvPHT*) or for regulators of the Gcv system (*gcvA* and *lrp*) (Table 1) (38). In the metabolite suppression assay, a *glyA* gene deletion was rescued by the addition of glycine (Table S3). Interestingly, the synthetic lethality observed was not dependent on the metabolism of glycine, but on the metabolism of tetrahydrofolate. GlyA and the Gcv system are the only two pathways for the recycling of tetrahydrofolate that can produce 5,10-methylene-tetrahydrofolate, which is required for many other cellular reactions (12).

Our synthetic interaction data are therefore enriched in interactions that correspond to redundant pathways that converge to a single metabolite. In cases such as pantothenate, the metabolism is rather simple, consisting of a biosynthesis pathway and a transport protein. In other cases, however, the metabolism is more complex and involved different redundant pathways or enzymes as well as many transport proteins. We speculate that many of the genetic interactions with uncharacterized genes or with genes of unrelated pathways hold similar relationships.

### Glutamine biosynthesis.

**FIG 4:**
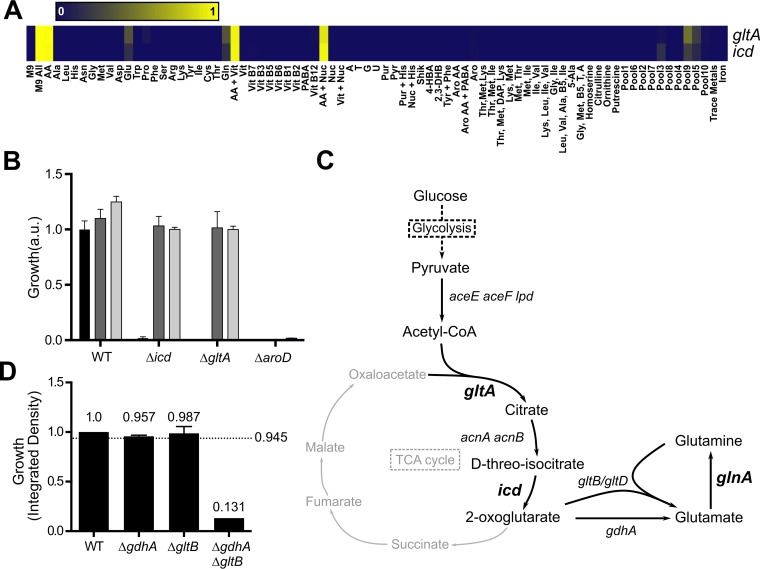
*icd* and *gltA* are part of the glutamate and glutamine biosynthesis pathway. (A) Metabolite suppression array of *icd* and *gltA* deletion mutants. The heatmap denotes gradient of growth (yellow) and growth inhibition (blue) in various nutrient conditions indicated as described by Zlitni et al. (22). M9 All, M9 minimal medium with all supplements: AA, all amino acids; Vit, vitamin; Nuc, all nucleobases; Pur, purines; Pyr, pyrimidines; Aro, aromatic amino acids; PABA, *para*-aminobenzoic acid. (B) The bar graph shows the impact of supplementation with glutamate and 2-oxoglutarate. *E. coli* strain BW25113 (WT) or kanamycin-resistant mutants with single gene deletions were grown in M9 minimal medium with glucose (M9-glucose) (black bars) supplemented with glutamate (100 µg/ml) (dark gray bars) or supplemented with 2-oxoglutarate (100 µg/ml) (light gray bars). The growth was normalized to the growth in M9-glucose (WT) or to the growth in M9 medium supplemented with glutamate (*icd* and *gltA*) or with shikimate (*aroD*; data not shown). Growth is shown in arbitrary units (a.u.). (C) Glutamate and glutamine biosynthesis pathway. Genes that are essential during growth in nutrient-limited media are indicated in boldface type and a larger font size. Acetyl-CoA, acetyl coenzyme A; *gltB*/*gltD*, *gltB* and *gltD*. (D) Multiplicative approach to identify a synthetic lethal interaction between *gdhA* and *gltB* in M9 minimal medium. The growth of each single deletion mutant and of the double deletion mutant was normalized to that of WT *E. coli* strain BW25113. The dotted line represents the expected growth of the double deletion mutant based on the accumulation of the single deletions.

While most nutrient stress genes are clearly linked to the biosynthesis of a particular nutrient, this is not the case for *icd* and *gltA*. Isocitrate dehydrogenase and citrate synthase, encoded by the genes *icd* and *gltA*, respectively, are two enzymes that are part of the tricarboxylic acid (TCA) cycle. They are the only two enzymes in the TCA cycle that are essential in nutrient-limited conditions, even though many intermediates in the TCA cycle are linked to amino acid biosynthesis (39). In the metabolite suppression assay, both *icd* and *gltA* deletion mutants are suppressed by glutamate, glutamine, or pools of metabolites containing either glutamate or glutamine (Fig. 4A; see Table S3 in the supplemental material). This suggested that they are involved in glutamate and glutamine biosynthesis. In fact, Icd and GltA enzymes are responsible for two of the first steps of the TCA cycle that lead to the formation of 2-oxoglutarate.

We tested whether supplementing with 2-oxoglutarate would rescue the growth of the Δ*icd* and Δ*gltA* mutants in M9 minimal medium. As hypothesized, 2-oxoglutarate rescued the growth of the Δ*icd* and Δ*gltA* mutants in M9 minimal medium, as did glutamate (Fig. 4B). We further observed this link between early steps of the TCA cycle and glutamate/glutamine biosynthesis in our synthetic interaction data. Indeed, *icd* and *gltA* formed synthetic sick or lethal interactions with genes encoding subunits of the glutamine or glutamate transporters (Table 1). These data suggest that the early steps of the TCA cycle participate in glutamate and glutamine biosynthesis when their availability is reduced. With the exception of the steps where more than one enzyme can perform the reaction, genes encoding components of the enzymatic processes in glutamate and glutamine biosynthesis are essential in nutrient-limited conditions (Fig. 4C). AcnA and AcnB are isozymes, and thus, the deletion of one of them is not enough to abolish the reaction. Similarly, we reasoned there must be another enzyme that catalyzes the transformation of 2-oxoglutarate into glutamate, since the *gdhA* gene is not essential for growth in M9 minimal medium. We therefore crossed a *gdhA* deletion mutant with the Keio collection and analyzed the growth of the double deletions in M9 minimal medium (see Table S2 in the supplemental material). The *gdhA* gene formed synthetic sick/lethal interactions with *gltB* and *gltD*, the two subunits of the glutamate synthase that uses glutamine to transform 2-oxoglutarate into glutamate (Fig. 4D). Interestingly, glutamate synthase can also utilize ammonia instead of glutamine *in vitro*, although with a lower affinity (40). These data suggest that glutamate synthase can also substitute for the loss of *gdhA* to convert 2-oxoglutarate into glutamate even in the absence of glutamine.

### *yigM* is the biotin transporter BioP.

Biotin transport in *E. coli* is catalyzed by an orphan transporter, where the coding gene has not yet been identified. Indeed, biotin is actively transported in *E. coli*, and this biotin transport activity has been assigned to the orphan protein BioP (41, 42). More than 40 years ago, the gene encoding the biotin transporter was mapped to a location between the *ilvC* and *metE* loci (42). Since biosynthesis genes often form synthetic lethal interactions with transporter genes, we asked whether it was possible to identify the gene responsible for the biotin transport function from our synthetic genetic array performed with a gene involved in the biosynthesis of biotin.

**FIG 5:**
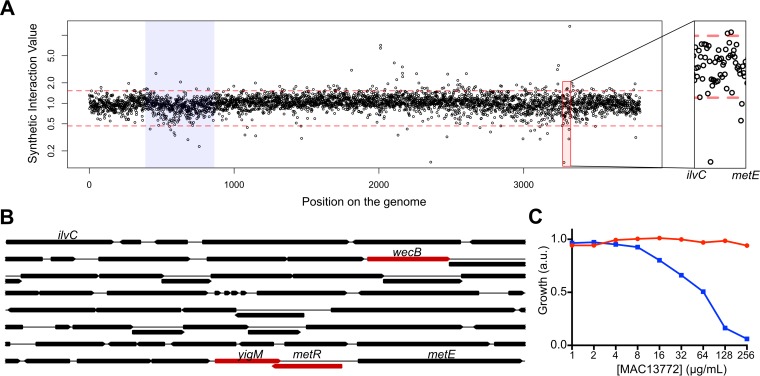
*yigM* encodes the biotin transporter BioP**.** (A) Index plot showing the synthetic interaction value of every double deletion mutant resulting from the mating of the *bioA* strain with the Keio collection. The region between the *ilvC* and *metE* genes is highlighted (right panel). (B) The genetic locus between the *ilvC* and *metE* genes. The genes that are involved in synthetic sick/lethal interactions with *bioA* within that locus are shown in red. (C) Effect of the BioA inhibitor MAC13772 on the growth of *E. coli* strain BW25113 (red) or the kanamycin-resistant *yigM* mutant (blue) in LB. The growth was normalized to that of LB with no drugs.

In our synthetic genetic array, *bioA* formed synthetic sick or lethal interactions with three genes present between the *ilvC* and *metE* genes: *wecB*, *yigM*, and *metR* (Fig. 5A and B). Of these three genes, only *yigM* was a gene of unknown function. Furthermore, only *yigM* is predicted to be an inner membrane protein and could potentially be a transporter. Interestingly, the *yigM* gene overlaps substantially with the *metR* gene (Fig. 5B). It is, therefore, likely that the synthetic lethal phenotype of the *metR-bioA* pair is due to the concomitant disruption of the *yigM* gene by the *metR* deletion mutant. In agreement with this hypothesis, a *metR* deletion mutant formed a synthetic lethal interaction with *bioA* (see Table S2 in the supplemental material). The *wecB* gene, involved in the biosynthesis of the enterobacterial common antigen, is involved in synthetic interactions across ~25% of the synthetic genetic array. Thus, *yigM* was the most likely candidate for the biotin transporter.

To verify the role of the *yigM* gene in biotin transport, we utilized a chemical genetic approach. MAC13772 inhibits the growth of *E. coli* in M9 minimal medium by inhibiting the enzymatic activity of BioA, a key enzyme in biotin biosynthesis (22). We reasoned that synthetic lethality between biosynthesis and transport would mean that MAC13772 should inhibit the growth of the *yigM* deletion mutant in rich media. As expected, MAC13772 inhibited the growth of the *yigM* deletion mutant in LB, but not that of wild-type (WT) *E. coli* BW25113 (Fig. 5C). MAC13772 also inhibited the growth of the *metR* deletion mutant, further confirming that the *metR* deletion also disrupted the *yigM* gene (see Fig. S6 in the supplemental material). Interestingly, *yigM* has also been previously linked to the transport of biotin (43). Therefore, *yigM* encodes the biotin transporter BioP and should be renamed *bioP*.

## DISCUSSION

Herein, we have defined the genetic network that responds to nutrient stress in *E. coli* using a synthetic genetic array approach to engineer a genome-scale cross of strains harboring deletions in some 82 nutrient stress genes with the entire *E. coli* gene deletion collection (Keio). An analysis of the growth of the resulting double deletion strains on rich microbiological media revealed an average of 23 synthetic lethal interactions for each nutrient stress gene. A large majority of these interactions was with genes of unknown function or with genes that have roles in unrelated pathways, indicating that the network defining nutrient stress is surprisingly complex. In total, the genetic interaction network reported here provides a quality data set to further mine for missing links in nutrient synthesis and to characterize additional genes of unknown function in *E. coli*. Ultimately, a better understanding of bacterial growth under nutrient stress could aid in the development of novel antibiotic discovery platforms. For example, the synthetic lethal interactions described here provide a large collection of target pairs that can be further explored with a strategy where combinations of compounds that target the nutrient stress network might lead to growth inhibition on rich media. Furthermore, the synthetic lethal interaction data set also has potential to facilitate mechanism of action studies of active compounds where idiosyncratic gene-gene interactions can be phenocopied with chemical-gene combinations.

The network described herein revealed remarkable metabolic robustness in the way of pathway redundancies and interactions between nutrient acquisition and biosynthesis. Indeed, bacteria often use more than one pathway to synthesize amino acids, nucleotides, and vitamins or to acquire these metabolites from the environment. We observed many synthetic interactions between genes that lead to a common metabolite. These synthetic interactions led us, for example, to better understand the glutamate/glutamine biosynthesis pathway and to identify the gene responsible for the transport of biotin. It is therefore probable that many synthetic interactions with genes of unknown function or genes from unrelated pathways hold similar relationships. Some of the most profound synthetic lethal interactions that we have observed were between biosynthetic and transport genes. This was the case for the biosynthesis of arginine, tyrosine, lysine, glutamate, and glutamine as well as vitamins. This suggests that these biosynthetic pathways are relatively simplistic, consisting of one biosynthetic route and one transporter, and are highly efficient. This contrasts with other pathways, such as branched-chain amino acid metabolism, where many transporters and different biosynthetic routes abound, and we found fewer such gene-gene interactions.

In bacteria, synthetic genetic arrays have typically used 384-colony density formats (25, 26, 44). To our knowledge, our work represents the first study describing the conjugation of bacteria at 1,536-colony density and arraying bacteria at 6,144-colony density, significantly increasing throughput of this approach. Moreover, artifactual interactions that are a consequence of the proximity of query and target gene deletions are typically dealt with by ignoring interactions that arise within 20 to 30 kb on either side of the target gene (26). Our analysis is capable of detecting synthetic lethal interactions in much closer proximity to the target gene by making use of the data in these regions with a rolling median. This rolling median analysis accounts for lower frequencies of recombination that are produced in these regions.

Recently, several groups have attempted to predict synthetic lethal gene pairs in *E. coli* and other pathogens using metabolic models (45–47), and many of the predicted interactions involve the 82 nutrient stress genes that we have tested experimentally. Our data have confirmed only a small number of the predicted lethal interactions, such as the interaction of *panF* with the pantothenate biosynthesis genes *panBCD*, *metL* with *thrA*, and the interaction between the NAD biosynthesis genes *nadABC* and the gene *pncB* from the NAD salvage pathway (45, 46). Most computationally predicted lethal interactions however did not show any synthetic phenotype. Given the large number of interactions with uncharacterized genes, it seems likely that, in many instances, the metabolic models were missing key information. One of the reasons for this is that metabolic models are limited by gene expression data and functional annotations, and they tend to overlook genes that are uncharacterized or that have been misannotated. For example, most metabolic models predict a synthetic lethal interaction between the molybdate transporter genes *modABC* and the sulfate/thiosulfate transporter genes *cysAUW*, since the sulfate/thiosulfate can act as an alternate molybdate transport system (45, 46, 48). In our synthetic array, we did not identify any interactions between the nutrient stress gene *cysU* and the *modABC* genes. Thus, it appears likely that molybdate transport is redundant, with a third unidentified transporter (48). In all, we conclude that, while metabolic models are useful for hypothesis generation, experimental data are crucial to identify synthetic lethal gene pairs. Indeed, our synthetic genetic array data raise many questions owing to an incomplete understanding of bacterial nutrient synthesis and represent a quality repository for computational approaches that will surely provide additional hypotheses for experimental validation.

Current antimicrobial drugs focus almost exclusively on a limited number of processes that have proven to be essential for the growth of bacteria in nutrient-rich conditions. Nevertheless, additional processes such as the synthesis of amino acids, vitamins, and nucleobases become essential when bacteria are grown *in vitro* on minimal microbiological media, and a variety of *in vivo* studies have suggested that nutrient stress may be a better proxy for infection conditions in a host. Indeed, sulfonamide drugs that target folate synthesis in bacteria have proven to be a very successful class of antibiotics (49). Work many years ago out of Stocker’s laboratory led to an understanding that enteric bacteria lacking the ability to synthesize aromatic amino acids were avirulent and made good vaccine strains (50). Further, a large number of *in vivo* genetic studies of bacterial virulence have implicated nutrient biosynthesis as a viable antibacterial target in a variety of pathogens (13, 51–54). Where the conventional essential gene set numbers just 303 in the model bacterium *E. coli* (1), those genes that are essential under nutrient stress (119 genes) offer the potential to considerably broaden the target base for antibiotic discovery. Success in targeting nutrient biosynthesis will come from a thorough understanding of both *in vivo* dispensability and of the genetic network that underpins nutrient biosynthesis in bacteria.

## MATERIALS AND METHODS

### Strains, gene deletions, and growth conditions.

*Escherichia coli* strain BW25113 [F^−^ Δ(*araD*-*araB*)*567 lacZ4787*Δ::*rrnB-3* LAM^−^ *rph-1* Δ(*rhaD*-*rhaB*)*568 hsdR514*] was used in this study for standard assays and to create single gene deletions by replacement of the gene by an apramycin resistance cassette. Alternatively, we used kanamycin-resistant single gene deletions from the Keio collection, a collection of all nonessential single gene deletions made in strain BW25113 (1). Bacteria were routinely grown at 37°C for 18 h in LB or M9-glucose and ampicillin (100 µg/ml), apramycin (100 µg/ml), spectinomycin (100 µg/ml), or kanamycin (50 µg/ml) if needed and unless stated otherwise.

Single gene deletions were made by homologous recombination (55, 56). Briefly, *E*. *coli* BW25113 was first transformed with the plasmid pSim6, containing the *exo*, *beta*, and *gam* genes from phage λ under the control of a temperature-sensitive promoter (55). Cells were then grown at 30°C to an optical density at 600 nm (OD_600_) of 0.8, and the expression of the λ genes was induced by a 20-min heat shock at 42°C. Finally, cells were made competent for electroporation, transformed with PCR products that consist of an apramycin resistance cassette flanked by 50-bp regions of homology to the targeted gene and plated on LB agar containing apramycin (100 µg/ml). The PCR products were generated by amplifying the apramycin resistance cassette from plasmid pSET152 previously linearized with PciI (New England Biolabs) (57). The PCR products were obtained using *Phusion* polymerase (Life Technologies, Inc.) and the apramycin amplification primers listed in Table S1 in the supplemental material with a melting temperature (*T*_*m*_) of 65°C and 45 s of elongation (1). Apramycin amplification primers contain a 50-bp homology region to the target gene followed by the sequence 5′-AGCAAAAGGGGATGATAAGTTTATC-3′ for the forward primer and the sequence 5′-TCAGCCAATCGACTGGCGAGCGG-3′ for the reverse primer. Recombinants were confirmed by two PCRs using primers upstream and downstream of the targeted gene (Table S1) and primers inside the apramycin cassette (primers F [5′-CAGAGATGATCTGCTCTGCCTG-3′] and R [5′-CAGGCAGAGCAGATCATCTCTG-3′]).

### Synthetic genetic interaction array.

The generation of double deletion mutants was achieved using synthetic genetic array technology (25, 26). Briefly, apramycin-resistant single gene deletions were first rendered competent for conjugation using pseudo-F^+^ *E. coli* strains containing a chromosomal integrative plasmid (CIP) that contains the machinery to allow for F conjugation (25). These CIP strains also contain a spectinomycin resistance cassette and are auxotrophic for diaminopimelate. There are 20 different versions of the CIP plasmids that integrate into the genome of *E. coli* at 10 different positions either in the clockwise or counterclockwise direction (58). To promote efficient mating, we used the CIP strain where the integration of the plasmid was the closest to the query gene. Overnight cultures of the apramycin-resistant deletion mutants and the appropriate CIP strains were cospotted together on LB agar containing 0.3 mM diaminopimelate in a 1:1 ratio and incubated overnight at 37°C. Hfr strains were then recovered by transferring the culture to a new LB agar plate containing both apramycin and spectinomycin.

To create double deletion mutants, the query mutations were transferred to every clone of the Keio collection by conjugation (see Fig. S1 in the supplemental material). First, each Hfr apramycin-resistant strain was arrayed at 1,536-colony density on LB agar containing apramycin using the Singer rotor HDA (Singer Instruments, United Kingdom). In parallel, the Keio collection was also arrayed at 1,536-colony density on LB agar plates containing kanamycin (three plates total). Using the Singer rotor HDA, the Hfr apramycin-resistant strain and the Keio collection from the 1,536-colony plates were then cotransferred onto LB agar plates without antibiotic selection, and the plates were incubated overnight at 30°C. Following incubation, the colonies were transferred to LB agar plates containing both apramycin and kanamycin to select for the double deletion mutants. Plates were incubated at 37°C for 18 h, and images were acquired every 20 min using high-quality scanners as previously described (27). The antibiotic selection did not have differential fitness effects on the different mutants (Fig. S2).

### Quantitative plate imaging and analysis.

Mating plates were imaged by the method of French et al. (27), using the normalization process described therein. Briefly, plates are scanned using Epson Perfection V750 transmissive scanners. Images were analyzed using ImageJ (59), and the amount of light absorbed by colonies during the transmissive scanning was quantified as integrated density, a value that tracks with cell number in a linear manner. Full details of the image acquisition and analysis are available in the article by French et al. (27). We further normalized our data to account for the expected growth of the double deletion mutant, which corresponds to the product of the growth of each single mutant compared to the growth of the WT. The “synthetic interaction value” (SIV) then corresponds to the ratio of the observed growth (“integrated densities”) to the expected growth. A value of 1 indicates that the mutant grows as expected, while a value of <1 is indicative of a synthetic sick or lethal interaction.

As others have noted (25), we observed that the proximity between the query gene deletion and the recipient gene deletion could create artificial synthetic lethal interactions. This occurs because the efficiency of recombination decreases when the genes are close or alternatively because the recipient antibiotic resistance cassette is flipped out during recombination. To counter this, we first ordered the genes based on their position on the chromosome to highlight a “dip” in the index plot and then identified the regions flanking the query gene deletion. Logarithmic curves were fit to the data coinciding with this “dip” on either side of the Hfr knockout (Fig. 1). The range of the logarithmic fit was determined by first examining the rolling median across the data for each individual treatment and then identifying when the gradual decrease in integrated density occurred. Symmetrical logarithmic curves were fit to this region, with the gene of interest at the cusp of the dip. Finally, the fit curves were set to 1, aligning the points affected by the dip to the rest of the data. Once data were aligned to 1, this provided a more typically Gaussian distribution that allowed us to compare treatments without artifactual synthetic lethal combinations based solely on proximity. Synthetic sick and lethal interactions were identified using a 2.5 standard deviation cutoff. We also recreated mutants with double deletions of genes in this region and confirmed the accuracy of the rolling median correction (see Fig. S3 in the supplemental material). Genetic interaction networks were prepared using the R statistical computing language (60) and BioLayout Express 3D (61) by the method of French et al. (27).

Data were also mined based on the Gene Ontology information available for synthetic lethal combinations, particularly their cellular process targets. All synthetic lethal interactions for each gene were assigned at least one GO term, and the number of times each GO term arose as a hit was compiled. In this manner, we are able to identify the general targets of synthetic crosses with the Keio collection and look for enrichment in unexpected cell processes.

## SUPPLEMENTAL MATERIAL

Text S1  Metabolic profiling of the nutrient stress genes. Download Text S1, DOCX file, 0.6 MB

Figure S1  Synthetic genetic array protocol. Hfr strains of the deletions of the nutrient-limited essential genes and the Keio collection were arrayed at 1,536-colony density on LB agar plates containing apramycin and kanamycin, respectively (step 1). The two plates were then combined by cospotting on LB agar plates without antibiotics (step 2). The mating plates were then transferred to the selection plates by spotting in quadruplicate on LB agar plates containing both apramycin and kanamycin (step 3). Finally, the growth of each colony was analyzed (step 4). Download Figure S1, TIF file, 1.6 MB

Figure S2  Growth of *argA* double deletion mutants with or without antibiotic selection. To verify the effect of the antibiotic selection on the growth of the different mutants, we have measured the growth of double deletions made with *argA* in the presence or absence of the antibiotic selection as presented in the article. Growth without antibiotic selection was done as follows: after the conjugation step, the mutants were spotted on a selection plate (containing apramycin and kanamycin) at 1,536-colony density and grown overnight. The double deletion mutants were then pinned on an LB plate without antibiotics in quadruplicate (to 6,144-colony density), and the growth was measured (as presented in the manuscript) after 18 h at 37°C. The antibiotic selection did not have a differential effect on the growth of specific mutants but rather had an effect that was generalized to all mutants. Download Figure S2, TIF file, 0.2 MB

Figure S3  Correction of the “dip” around the query gene deletion. To verify that the rolling median approach for correcting the “dip” was accurate, we recreated double deletion mutants that were closely linked to the *argA* gene. The synthetic interaction values shown here are those of the synthetic interaction array before the rolling median correction (black bars), after the correction (red bars) and those of the recreated double deletions (blue bars). Download Figure S3, TIF file, 0.1 MB

Figure S4  Gene Ontology analysis of the synthetic genetic array. Gene Ontology (GO) term overviews for synthetic lethal pairings with high frequency in the minimal essential genetic interaction array. Occurring with highest frequency are transport, metabolism, and redox processes. GO terms were assessed using pathway tools in EcoCyc (2). Download Figure S4, TIF file, 0.8 MB

Figure S5  Gene interactions in pantothenate biosynthesis. (A) Network map of the synthetic sick and lethal gene pairs formed with the pantothenate biosynthesis genes *panB*, *panC*, *panD*, and *panZ*. Pantothenate biosynthesis genes as well as the common interacting genes are highlighted in red. (B) Pantothenate biosynthesis pathway showing the nutrient-limited essential genes and the pantothenate transporter *panF*. (C) Table showing the synthetic lethal genes from the cross of a *panF* deletion mutant with the Keio collection. Download Figure S5, TIF file, 1.8 MB

Figure S6  Activity of MAC13772 against a *metR* deletion mutant. *E. coli* strain BW25113 and the *yigM* and *metR* deletion strains were grown with or without 256 µg/ml of the BioA inhibitor MAC13772. Relative growth between untreated and treated bacteria is shown. Download Figure S6, TIF file, 0.1 MB

Table S1  List of primers used in this study. Apramycin amplification primers were used to create the deletions in the nutrient stress genes, and the confirmation primers were used to confirm the deletions. Table S1, XLSX file, 0.05 MB

Table S2  Synthetic interaction values of the double deletion mutants. Synthetic interaction values were calculated for the double deletion mutants as explained in Materials and Methods. The growth of the double deletions for the nutrient stress genes and the *panF* gene was measured in LB, while the growth of the *gdhA* mutant was measured in M9-glucose. Table S2, XLSX file, 2.4 MB

Table S3  Metabolic suppression array of the nutrient stress genes. Keio clones were grown in M9-glucose minimal medium in the presence or absence of metabolites or pools of metabolites and normalized to growth in M9All. Amino acids are referred to by their three-letter code, and nucleobases are referred to by their one-letter code. All, all supplements; AA, all amino acids; Nuc, all nucleobases; Pur, purines; Pyr, pyrimidines; Shik, shikimate; 4-HBA, 4-hydroxybutyl acrylate; 2,3-DHB, 2,3-dihydroxybenzoic acid; Aro, aromatic amino acids. Table S3, XLSX file, 0.1 MB

Table S4  Synthetic sick and lethal interactions. List of synthetic sick and lethal interactions identified in this study between the nutrient stress genes (query gene) and the Keio gene deletion (synthetic interaction partners). Table S4, XLSX file, 0.1 MB
