# Author Correction for Côté et al., The Genome-Wide Interaction Network of Nutrient Stress Genes in *Escherichia coli*

**DOI:** 10.1128/mBio.02138-16

**Published:** 2016-12-20

**Authors:** Jean-Philippe Côté, Shawn French, Sebastian S. Gehrke, Craig R. MacNair, Chand S. Mangat, Amrita Bharat, Eric D. Brown

**Affiliations:** Michael G. DeGroote Institute for Infectious Disease Research, Department of Biochemistry and Biomedical Sciences, McMaster University, Hamilton, Ontario, Canada

## AUTHOR CORRECTION

Vol. 7, no. 6, doi: 10.1128/mBio.01714-16, 2016. We report herein two referencing oversights in this article. We wish to emphasize here the important work by Ringlstetter (1), who first linked *yigM* to biotin transport in *Escherichia coli*. Our article overlooked the discovery of *yigM* as the coding gene for biotin transporter. Overlooked also was the work of Finkenwirth et al. (2), who created a Δ*yigM* Δ*bioH* deletion mutant (*E. coli*) and characterized biotin transporters from various organisms. These corrections do not impact the integrity of the data presented, nor are the dominant conclusions of the study altered.
