# Supplementary material for: The Genome-Wide Interaction Network of Nutrient Stress Genes in Escherichia coli
Source: mBio. 2016 Nov 22;7(6):e01714-16. doi: 10.1128/mBio.01714-16 (PMC5120140; doi:10.1128/mBio.01714-16)
Supplement: Text S1 — Metabolic profiling of the nutrient stress genes. Download [file mbo006163075s1.docx]

**Supplementary results – Metabolic profiling**

**The genome-wide interaction network of nutrient stress genes in *Escherichia coli*.**

Jean-Philippe Côté ^*^, Shawn French ^*^, Sebastian S Gehrke, Craig R MacNair, Chand S Mangat, Amrita Bharat and Eric D Brown ^#^

Michael G. DeGroote Institute for Infectious Disease Research, Department of Biochemistry and Biomedical Sciences, McMaster University, Hamilton, Ontario, Canada

^#^ Corresponding author: ebrown@mcmaster.ca

^*^ These authors contributed equally to this work

**Method**

Metabolic profiling was carried out on the nutrient-limited essential gene deletions as previously described (1). Metabolic profiling consists in supplementing minimal media with single or pools of metabolites; pools of metabolites are described in *Zlitni et al.* (1). Since the nutrient-limited essential gene deletions do not grow in M9 minimal media, careful preparation of the cultures must be done in order to prevent carryover of metabolites. Briefly, overnight cultures of the nutrient-limited essential gene deletions from the Keio collection were sub-cultured in 5 mL LB to an OD_600_ of ~0.6. At this point, the cells were washed 3 times with M9 media and resuspended in 5 mL of M9 media. The cells were then incubated for 1h at 37ºC and the OD_600_ was adjusted to 0.5.

The supplements (2.5uL/well, 20x stock solutions) were dry loaded to a 384-well plate and the strains were added (47.5uL/well; 1000-fold dilution in M9 media). The experiments were carried out in duplicates with one replicate in quadrant 1 and 2, and the other in quadrant 3 and 4 of the 384-well plate. The cells were incubated for 18h and the growth was monitored by measuring OD at 600nm. The growth was normalized to the M9 (low growth) and M9all (high growth) controls.

**Result and Discussion**

Metabolic suppression consists in supplementing minimal media with single metabolites or pools of metabolites that are absent from the nutrient-limited growth media. The idea is that by providing metabolites that are downstream of the steps mediated by the product of the nutrient-limited essential genes, we can rescue growth of these mutants in nutrient-limited conditions. In the array, different metabolites, amino acids, nucleotides and vitamins are added one by one or in pools (1). This metabolite suppression array has been helpful in the early step towards the determination of the mechanism of action of molecules that inhibits the growth of *E. coli* in minimal media (1).

As expected, growth defect of these 119 nutrient-limited essential genes was observed in M9-glucose media and was suppressed when the mutants were grown in M9-all, where all the nutrients were added to the M9-glucose (Fig. SR1). In general, gene deletions in the biosynthesis of a particular metabolite grew well when this metabolite was provided in the media. For instance, growth defects induced by the deletion of the genes involved in arginine biosynthesis were suppressed when arginine was provided in the M9 minimal media (Fig. SR2A). Moreover, the metabolite suppression array can discriminate between different steps of a same pathway. In arginine biosynthesis, *argA*, *argB*, *argC*, *argE*, *argG* and *argH* are essential for growth on minimal media (Fig. SR2B): *argA-E* leads to the formation of ornithine, which is then transformed in citrulline, and finally citrulline is transformed in arginine by *argGH*. As expected, ornithine and citrulline rescued the growth of the *argA-E* deletion mutant, but not the growth of the *argGH* mutant (Fig. 2A). Similarly, growth defect in pyrimidine, histidine and pantothenate biosynthesis genes for instance were suppressed by the addition of pyrimidine, histidine and pantothenate respectively.

The metabolite suppression array has identified conditions that rescued the growth of almost every gene deletion of the 119 nutrient-minimal essential genes. In some cases, only pools of metabolites were able the suppress the growth defect of the nutrient-limited essential genes. For instance, growth defect *aro* genes that are responsible for the biosynthesis of aromatic amino acids through chorismate were suppressed only when all aromatic amino acids were provided.

The metabolite suppression array has however failed to identified conditions that rescued the growth defect of the *ptsI* gene. *ptsI* is part of the phosphoenolpyruvate (PEP):carbohydrate phosphotransferase system (PTS) that is responsible for the transport and the phosphorylation of many carbohydrates in *E. coli* (2). The PTS system transfers the phosphate group from PEP to the carbohydrate using a cascade of enzymes and this is carried simultaneously with the transport of the carbohydrate in the cytoplasm of the cell. PtsI catalyzes the first step of the process and transfers the phosphate from PEP to the second enzyme in the pathway. Therefore, growth defects from the *ptsI* mutant is most likely due to the impossibility of the glucose to get inside the cells. Therefore, no metabolite in the array could suppress the growth defect of the *ptsI* mutant.

**Reference**

1. **Zlitni S**, **Ferruccio LF**, **Brown ED**. 2013. Metabolic suppression identifies new antibacterial inhibitors under nutrient limitation. Nature Publishing Group **9**:796–804.

2. **Postma PW**, **Lengeler JW**, **Jacobson GR**. 1993. Phosphoenolpyruvate:carbohydrate phosphotransferase systems of bacteria. Microbiol Rev **57**:543–594.


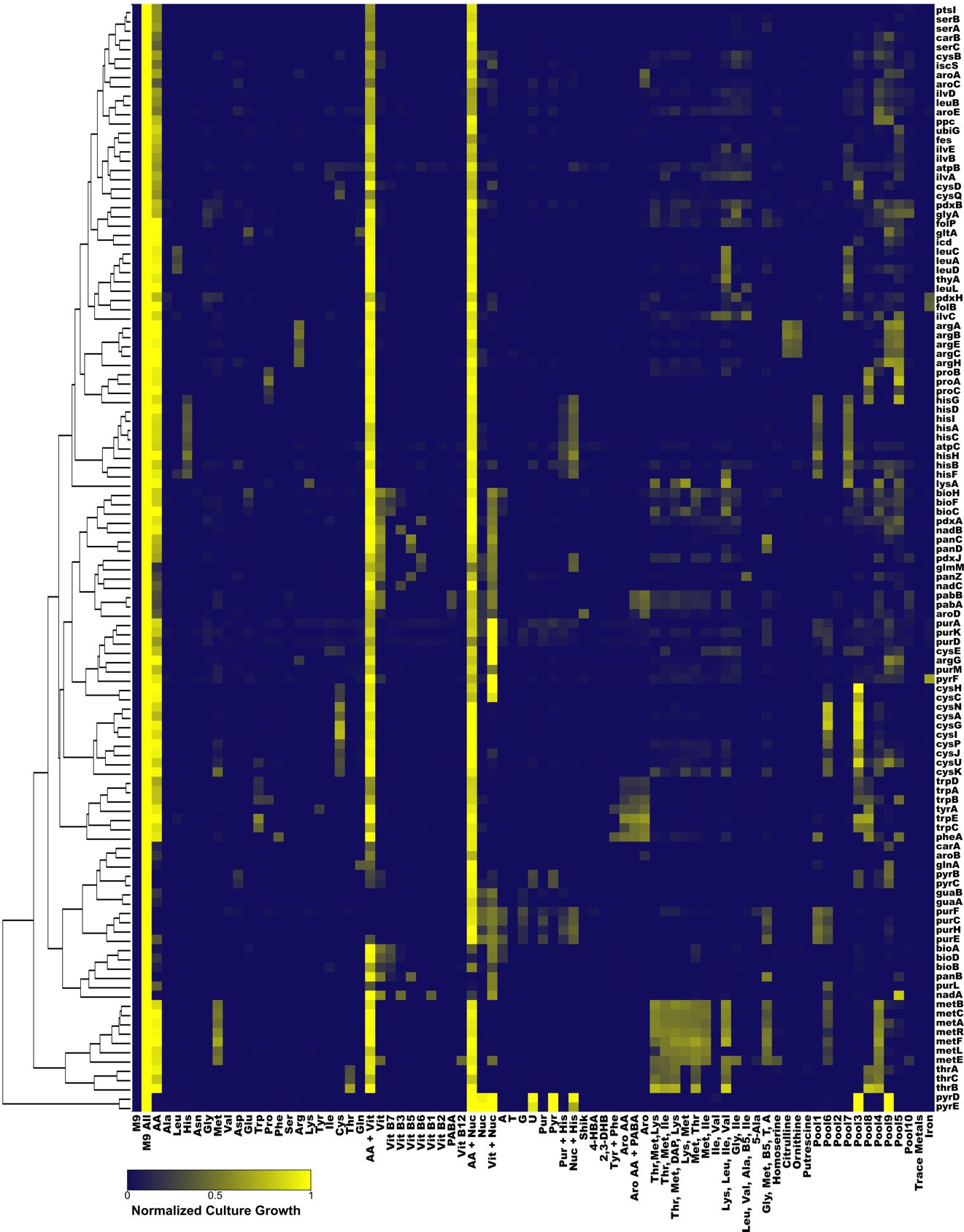


**Figure 1: Metabolite suppression array of the 119 nutrient-limited essential genes** Keio clones were grown in M9-glucose minimal media in the presence of absence of metabolites or pools of metabolites. Amino acids are referred to by their three-letter code and nucleobases by their one-letter code. All, all supplements; AA, all amino acids; Nuc, all nucleobases; Pur, purines; Pyr, pyrimidines; Shik, shikimate; 4-HBA, ; 2,3-DHB, ; Aro, aromatic amino acids.


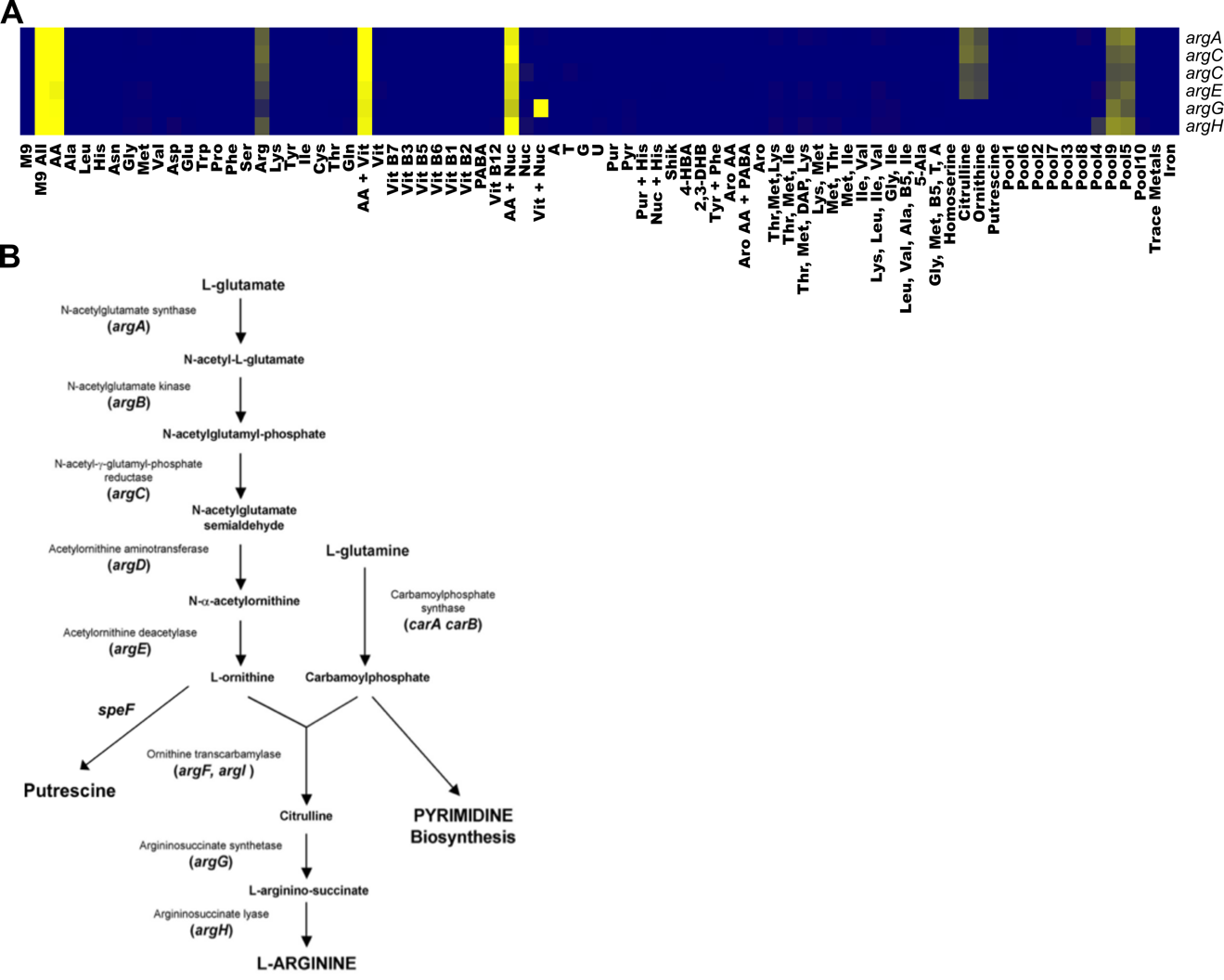


**Figure 2: Metabolite suppression array of arginine biosynthesis genes.**

(A) The metabolite suppression array for the nutrient-limited essential genes involved in the biosynthesis of arginine. *argGH* are not suppressed by citrulline and ornithine. (B) Arginine biosynthesis pathway.
