# Supplementary figures and images for: The Genome-Wide Interaction Network of Nutrient Stress Genes in Escherichia coli
Source: mBio. 2016 Nov 22;7(6):e01714-16. doi: 10.1128/mBio.01714-16 (PMC5120140; doi:10.1128/mBio.01714-16)

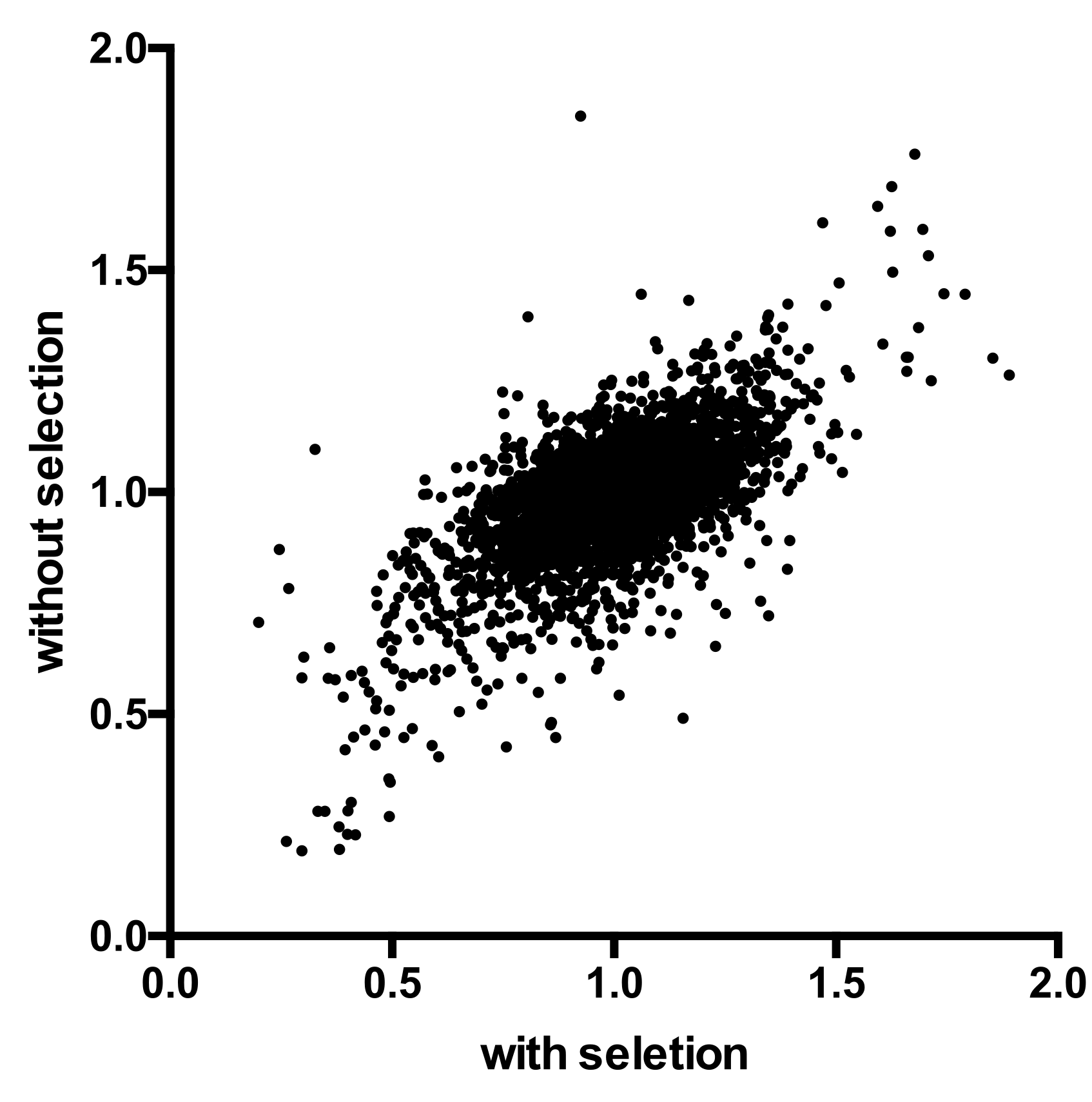

Supplement: Figure S2 — Growth of argA double deletion mutants with or without antibiotic selection. To verify the effect of the antibiotic selection on the growth of the different mutants, we have measured the growth of double deletions made with argA in the presence or absence of the antibiotic selection as presented in the article. Growth without antibiotic selection was done as follows: after the conjugation step, the mutants were spotted on a selection plate (containing apramycin and kanamycin) at 1,536-colony density and grown overnight. The double deletion mutants were then pinned on an LB plate without antibiotics in quadruplicate (to 6,144-colony density), and the growth was measured (as presented in the manuscript) after 18 h at 37°C. The antibiotic selection did not have a differential effect on the growth of specific mutants but rather had an effect that was generalized to all mutants. Download [file mbo006163075sf2.tif]

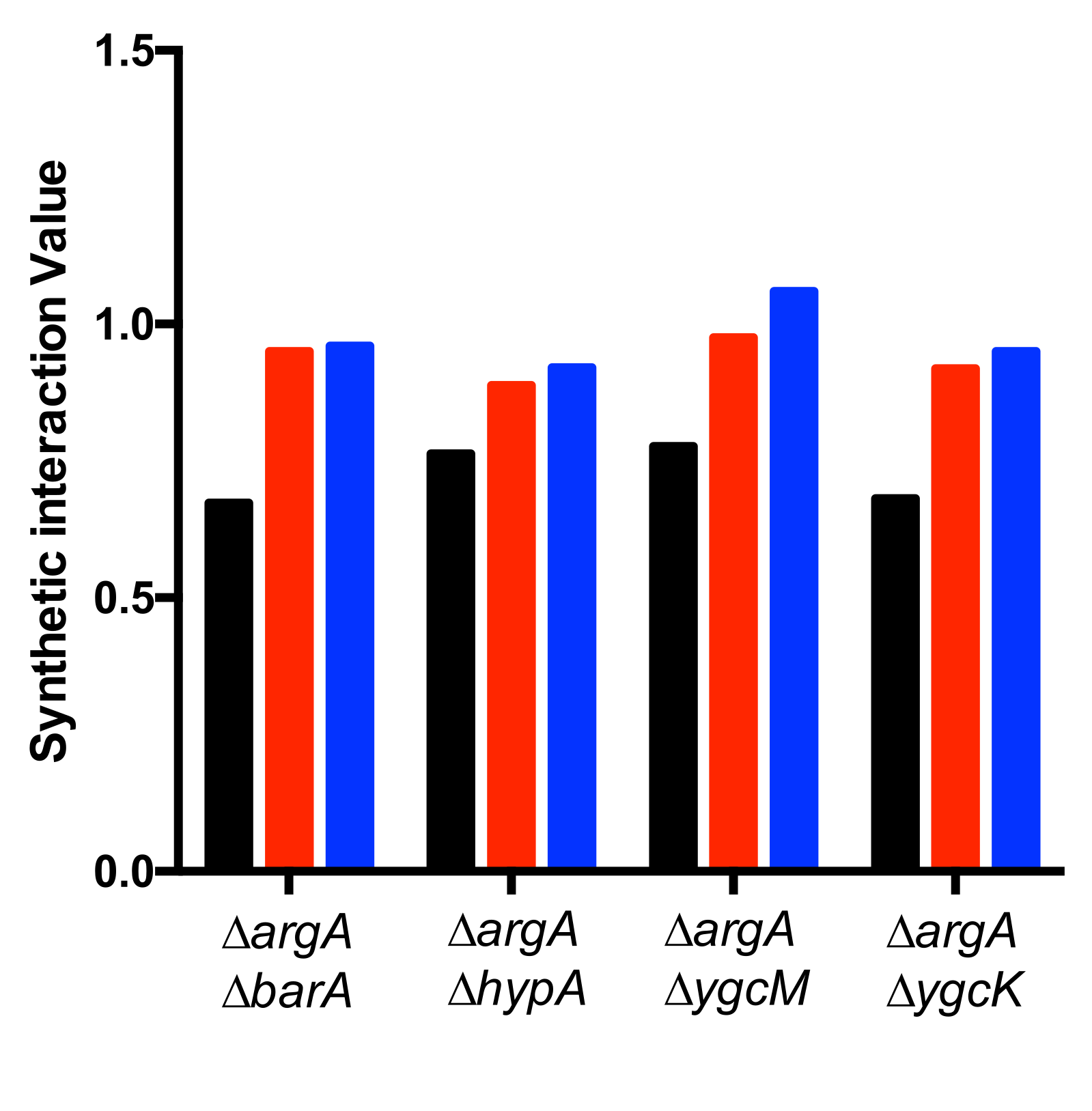

Supplement: Figure S3 — Correction of the “dip” around the query gene deletion. To verify that the rolling median approach for correcting the “dip” was accurate, we recreated double deletion mutants that were closely linked to the argA gene. The synthetic interaction values shown here are those of the synthetic interaction array before the rolling median correction (black bars), after the correction (red bars) and those of the recreated double deletions (blue bars). Download [file mbo006163075sf3.tif]

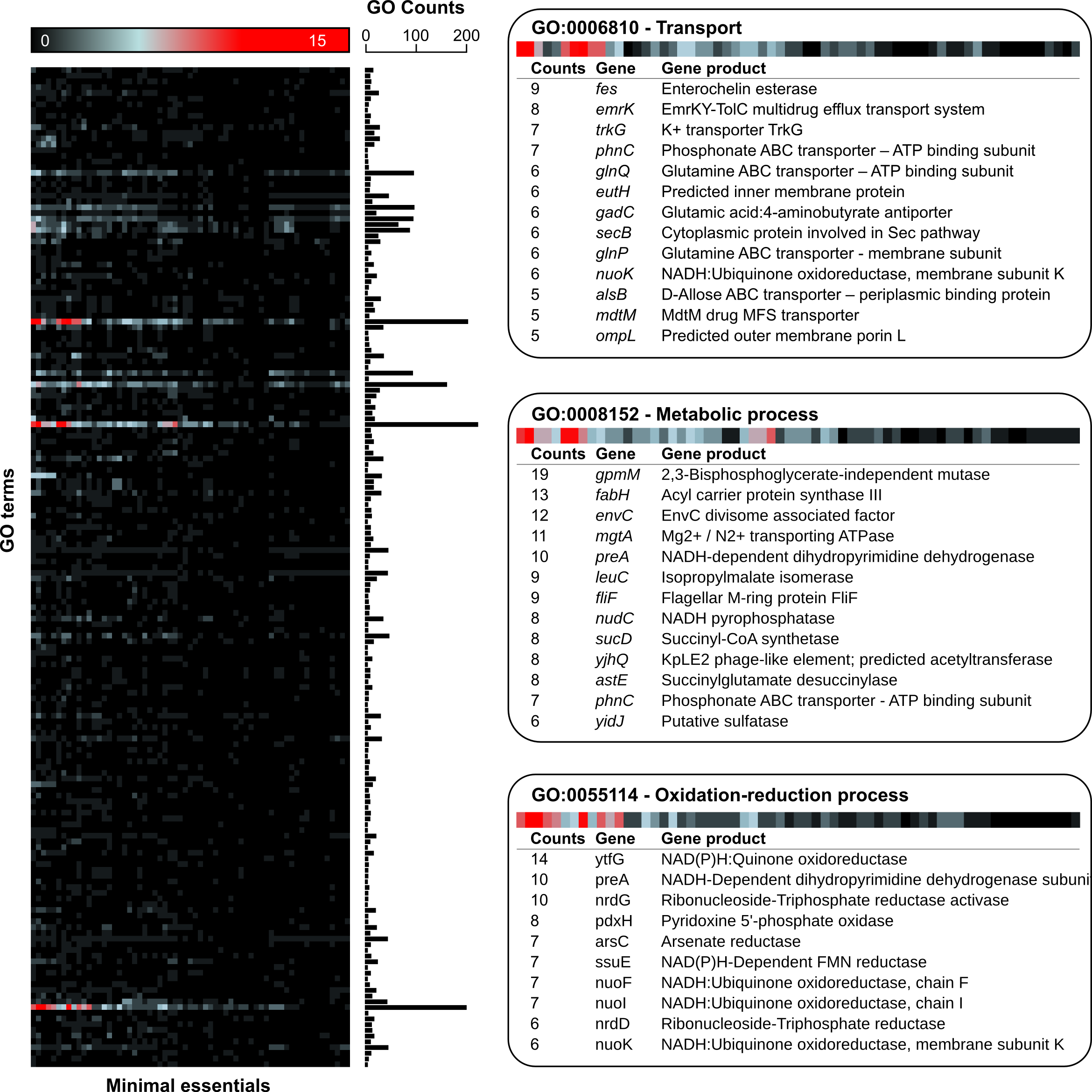

Supplement: Figure S4 — Gene Ontology analysis of the synthetic genetic array. Gene Ontology (GO) term overviews for synthetic lethal pairings with high frequency in the minimal essential genetic interaction array. Occurring with highest frequency are transport, metabolism, and redox processes. GO terms were assessed using pathway tools in EcoCyc (2). Download [file mbo006163075sf4.tif]

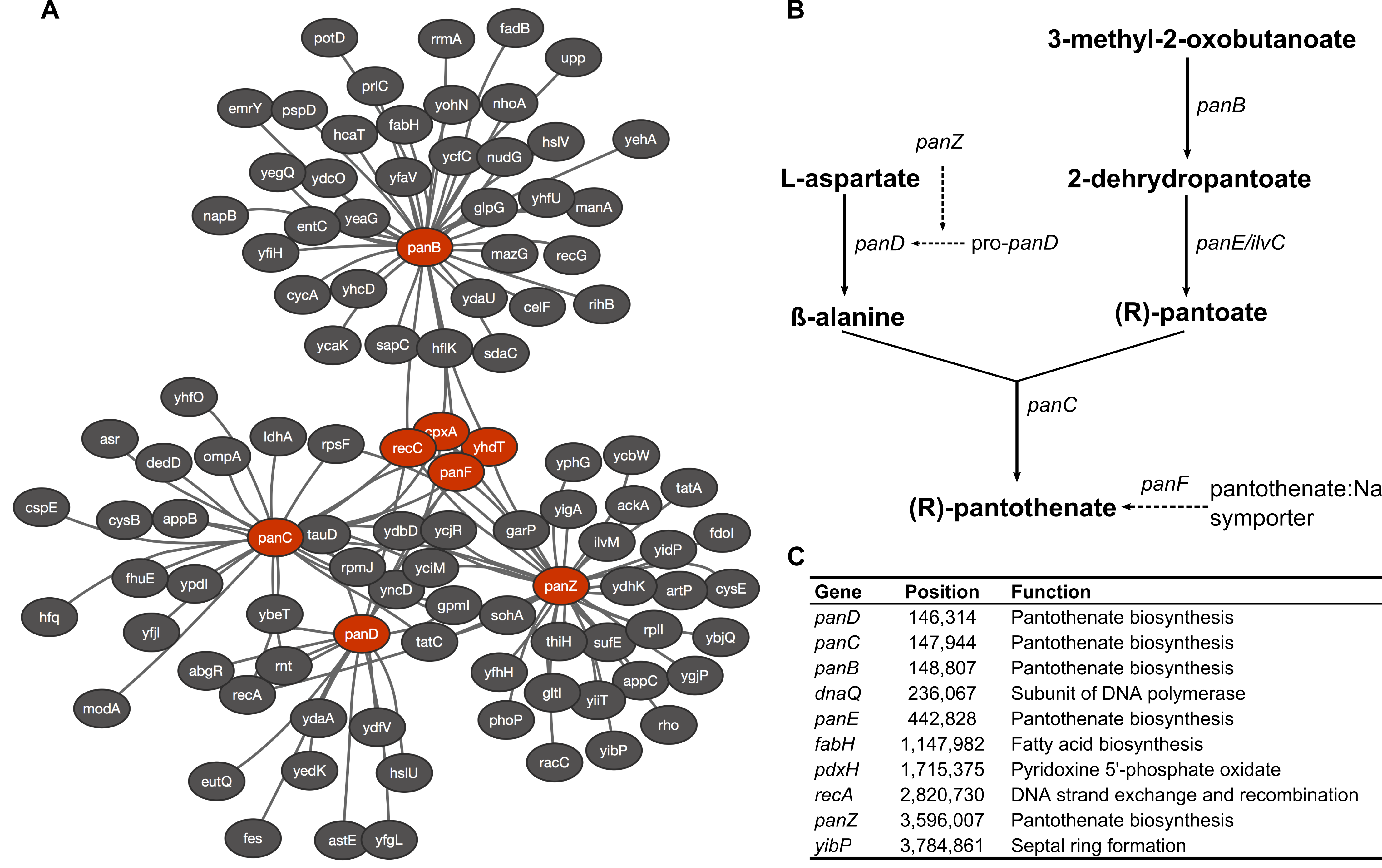

Supplement: Figure S5 — Gene interactions in pantothenate biosynthesis. (A) Network map of the synthetic sick and lethal gene pairs formed with the pantothenate biosynthesis genes panB, panC, panD, and panZ. Pantothenate biosynthesis genes as well as the common interacting genes are highlighted in red. (B) Pantothenate biosynthesis pathway showing the nutrient-limited essential genes and the pantothenate transporter panF. (C) Table showing the synthetic lethal genes from the cross of a panF deletion mutant with the Keio collection. Download [file mbo006163075sf5.tif]

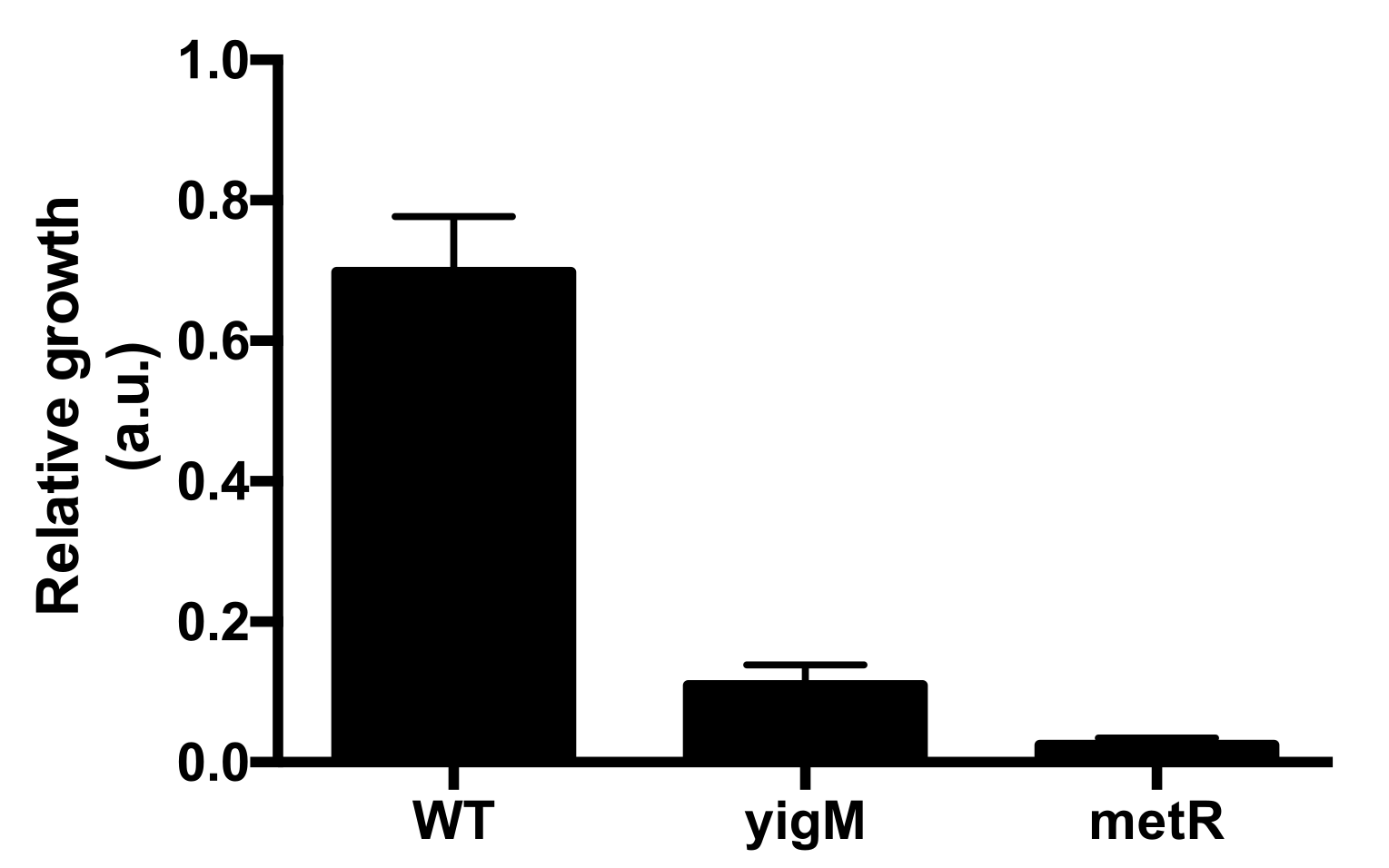

Supplement: Figure S6 — Activity of MAC13772 against a metR deletion mutant. E. coli strain BW25113 and the yigM and metR deletion strains were grown with or without 256 µg/ml of the BioA inhibitor MAC13772. Relative growth between untreated and treated bacteria is shown. Download [file mbo006163075sf6.tif]
